# Incidence and predictors of discrepancies in radiology resident interpretations of coronary CT in the emergency department

**DOI:** 10.1186/s12880-025-01781-3

**Published:** 2025-07-01

**Authors:** Na Young Kim, Ji Hoon Kim, Young Joo Suh

**Affiliations:** 1 Department of Radiology, Research Institute of Radiological Science, Severance Hospital, Yonsei University College of Medicine, 50-1 Yonsei-ro, Seodaemun-gu, Seoul, 03722 Republic of Korea; 2 Department of Emergency Medicine, Severance Hospital, Yonsei University College of Medicine, 50-1 Yonsei-ro, Seodaemun-gu, Seoul, 03722 Republic of Korea; 3 Institute for Innovation in Digital Healthcare, Yonsei University, 50 Yonsei-ro, Seodaemun-gu, Seoul, 03722 Republic of Korea

**Keywords:** Acute chest pain, Coronary computed tomography angiography, Emergency department, Discrepancy, Radiology resident

## Abstract

**Background:**

Discrepancies between preliminary reports by on-call radiology residents and final reports of coronary computed tomography angiography (CCTA) in the emergency department (ED) have not been thoroughly investigated.

**Methods:**

We conducted a retrospective quality assurance analysis of CCTA examinations performed during off-hours in a level-1 ED at a tertiary teaching hospital between March 2020 and April 2022. Discrepancies in identifying significant coronary artery disease (≥ 50% stenosis) between preliminary reports by on-call residents and final reports by board-certified cardiac radiologists were evaluated.

**Results:**

Among the 766 patient visits (median age, 59 years [interquartile range, 47–70]; 415 men), 82 cases (10.7%) showed discrepancies. Univariable logistic regression analyses identified HEART score, day of ED visit, ED crowding index, and coronary artery calcium (CAC) score as significant factors associated with discrepancies. Multivariable analysis revealed that an ED crowding index < 40 (adjusted odds ratio = 2.06; *P* = 0.005), and positive CAC scores were independently associated with increased discrepancies (adjusted odds ratio = 4.56 for scores > 0 and ≤ 100, *P* < 0.001; 4.79 for scores > 100 and ≤ 400, *P* < 0.001; 3.69 for scores > 400, *P* = 0.002). The rate of unnecessary invasive coronary angiography was significantly higher in the discrepancy group (80.0%, 12 of 15) compared to the agreement group (14.4%, 16 of 111) (*P* < 0.05).

**Conclusions:**

A substantial discrepancy rate was observed between preliminary and final CCTA interpretations in the ED. A lower ED crowding index and positive CAC scores were independently associated with an increased risk of discrepancies.

## Background

Coronary computed tomography angiography (CCTA) is a readily available, noninvasive anatomical test for patients presenting with acute chest pain in the emergency department (ED). It facilitates timely assessment of the presence and severity of coronary artery disease (CAD), supporting expedited patient disposition and management. Accordingly, for intermediate-risk patients with acute chest pain and no known CAD, CCTA received a Class I recommendation for ruling out atherosclerotic plaque and obstructive CAD in the 2021 American Heart Association/American College of Cardiology (AHA/ACC) guideline [1]. Patients with no or non-significant CAD (< 50% stenosis) on CCTA can generally be discharged safely from the ED, whereas those with significant CAD (≥ 50% stenosis) typically require hospital admission, treatment for acute coronary syndrome, and subsequent invasive coronary angiography (ICA) [2]. Thus, the timely and accurate interpretation of CCTA is essential for optimal patient care in the ED.

In most academic centers, radiology residents are responsible for preliminary interpretation of ED imaging studies during off-hours. In this setting, diagnostic errors can occur due to internal factors such as limited experience in cardiac imaging and fatigue, as well as external factors like backlogs related to ED crowding [3].

Discrepancy rates between preliminary reports by radiology residents and final reports by attending radiologists are commonly used as a surrogate marker for diagnostic errors among radiology trainees [4]. Previous studies have reported major discrepancy rates ranging from 0.1 to 10% across various diagnostic imaging modalities [2, 4–17]. However, limited research has specifically addressed CCTA interpretation in the ED setting [4–17]. Understanding the causes of diagnostic errors in CCTA is crucial, as such errors may lead to delayed or unnecessary ICA and inappropriate patient discharge. Therefore, the aim of this study was to evaluate the discrepancy rate between preliminary and final reports of CCTA performed in the ED and to identify factors associated with these discrepancies.

## Methods

### Study population

This study was a retrospective quality assurance analysis of CCTA examinations conducted in the level-1 ED at Severance Hospital, a tertiary teaching hospital located in Seoul. The primary objective was to evaluate interpretative discrepancies between preliminary and final CCTA reports during off-hours, in order to identify factors associated with reporting errors and inform improvements in ED imaging workflows. The study was approved by the institutional review board of Severance Hospital, which waived the requirement for informed consent (IRB 4-2023-0702).

From March 2020 to April 2022, a total of 815 adult patients (≥ 19 years old) with acute chest pain underwent CCTA during off-hours. Among these, 49 patients were excluded for the following reasons: prior coronary artery bypass graft surgery or stent insertion (*n* = 3), and missing medical records or preliminary CCTA reports (*n* = 46). These exclusions resulted in a final sample of 766 patients.

Part of the study population (509 of 766 patients) overlapped with a prior retrospective study that focused on comparing clinical outcomes of patients undergoing CCTA using a single-source versus a third-generation dual-source CT scanner [18].

### ED protocol for patients with acute chest pain

In our emergency department protocol, CCTA is performed in patients presenting with acute chest pain who are clinically assessed to be at low to intermediate risk for acute coronary syndrome. When the radiologist interprets the CCTA as showing significant coronary artery disease (≥ 50% stenosis), the emergency physician promptly consults cardiology for further evaluation and intervention. If no significant stenosis is identified, the patient is typically discharged with alternative diagnoses considered. During off-hours, clinical decisions are based on the preliminary interpretations by radiology residents.

### Resident on-call schedules and CT reporting process

At our institution, off-hours were defined as weekday evenings and overnights (6:00 PM to 8:00 AM), as well as weekends and public holidays. During off-hours, second- or third-year radiology residents provided preliminary real-time interpretations of CCTA examinations. A total of 21 residents participated in the on-call pool, each having completed at least two one-month cardiovascular imaging rotations and having interpreted approximately 150 CCTA studies per rotation.

Final reports were issued the following morning by one of nine board-certified cardiac radiologists, each with 5 to 23 years of experience in CCTA interpretation. For examinations performed on Fridays, weekends, or holidays, the final confirmation of the preliminary report was completed on the next working day (typically Monday).

### CCTA scan protocol

Two types of CT scanners were used in this study. A total of 280 patients underwent CCTA with a 64-detector row single-source CT scanner (Revolution EVO, GE Healthcare, Chicago, IL, USA). In this group, an oral β-blocker (50 mg of metoprolol tartrate) was administered prior to the scan in patients with a heart rate > 65 bpm, unless contraindicated.

The remaining 486 patients underwent CCTA with a third-generation dual-source CT scanner (SOMATOM Force, Siemens Healthineers, Forchheim, Germany) without administration of an oral β-blocker. Examinations were performed using either prospective or retrospective electrocardiogram (ECG)-gating, as appropriate. Detailed scan parameters have been described previously [18].

### CCTA report and data collection

All preliminary and final CCTA reports were written in accordance with the Coronary Artery Disease Reporting and Data System (CAD-RADS) [19]. The CAD-RADS category of each examination and any discrepancies between preliminary and final reports regarding the presence of significant CAD (≥ 50% stenosis) were investigated. At our institution, preliminary reports are stored in the electronic medical record system, allowing retrospective review even after modifications have been made in the final reports.

Coronary artery calcium (CAC) scores were calculated from non-contrast ECG-gated scans using the Agatston score [20]. Overall image quality was assessed by using a previously described five-point scale [21]: Excellent, no motion artifacts; Good, minor artifacts and mild blurring; Fair, moderate artifacts and moderate blurring without discontinuity; Poor, severe artifacts, doubling or discontinuity in the course of the coronary segments; or Unassessable, vessel structures not differentiable.

### Clinical data collection

Patients’ clinical data, including day and time of ED visits and the ED crowding indices, were collected from the electronic medical records.

According to The American College of Emergency Physicians (ACEP) Crowding Resources Task Force, ED overcrowding is defined as “a condition in which the identified need for emergency care exceeds available resources in the ED” [22]. Among various crowding indices described in the literature, we used an automated ED-specific metric that reflects the number of patients present in the ED at the time of the patient’s arrival [23].

Patients’ HEART scores were calculated based on history, ECG, age, risk factors, and troponin levels and were categorized into three risk groups: low (0–3), moderate (4–6), and high (7–10) [24].

The proportion of patients undergoing ICA and percutaneous coronary intervention (PCI) was analyzed. An ICA was considered “appropriate” if significant stenosis (≥ 50%) was reported in the preliminary report and subsequently confirmed on ICA. Conversely, an ICA was classified as “unnecessary” if significant stenosis was reported in the preliminary interpretation, but ICA revealed no or non-significant stenosis. Additionally, major adverse cardiovascular events (MACE) within 30 days of CCTA–including cardiac death, unexpected nonfatal myocardial infarction, and hospitalization for unstable angina–were recorded.

### Statistical analysis

Categorical variables were expressed as frequencies and percentages and compared using Chi-squared tests or Fisher’s exact test. Continuous variables were presented as medians with interquartile ranges (IQRs) and compared using the Mann-Whitney U test.

Univariable and multivariable logistic regression analyses were used to evaluate factors associated with discrepancies. Continuous variables were converted into categorical variables for regression analysis. Acquisition time was categorized into daytime (7:00 AM to 6:00 PM) and nighttime (6:00 PM to 7:00 AM). CAC scores were stratified into four categories based on widely used cutoffs: 0, > 0 and ≤ 100, >100 and ≤ 400, and > 400 [25]. Variables with *P* < 0.05 in univariable analysis were included in the multivariable model. Odds ratios (ORs) and 95% confidence intervals (CIs) were calculated. Multicollinearity was assessed using the generalized variance inflation factor. Model performance was evaluated by the area under the receiver operating characteristic curve (AUC).

In addition, we calculated the overall sensitivity, specificity, positive predictive value, negative predictive value, and accuracy of both preliminary and final reports using ICA results as the reference standard. Comparisons between the two report types were performed using the generalized estimating equation with an exchangeable working correlation structure. All statistical analyses were performed by using MedCalc version 20.019 (MedCalc Software, Ostend, Belgium) and R version 3.6.2 (R Foundation for Statistical Computing, Vienna, Austria). A *P* value < 0.05 was considered to indicate statistical significance.

## Results

### Discrepancy rates and baseline characteristics

A total of 766 patients were included in the study (median age, 59 years [IQR, 47–70]; 415 men [54.2%]).

**Fig. 1:**
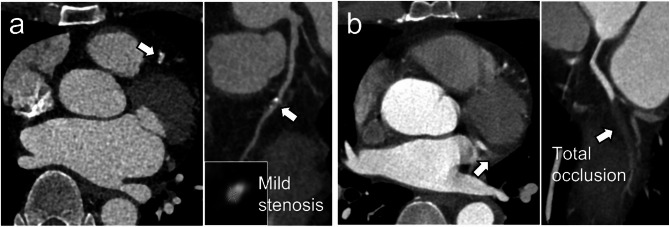
Cases of discrepancy

The discrepancy rate between the preliminary and final reports on CCTA was 10.7% (82/766). Among these discrepancies, radiology residents missed or underestimated significant CAD (≥ 50% stenosis) in 34 cases (41.5%), while they overestimated non-significant CAD in 48 cases (58.5%). Figure 1 presents representative cases of discrepancy.

Trans-axial (left) and curved planar reformation (right) images for each case. (a) Total occlusion at the obtuse marginal branch (arrows) was overlooked in the preliminary report. The emergency department (ED) crowding index was 21. The patient underwent percutaneous coronary intervention two days after the ED visit. (b) The on-call resident overestimated mild stenosis at the left anterior descending artery (arrows), interpreting it as significant (≥ 50%). Subsequent invasive coronary angiography revealed 40% stenosis at the corresponding site. The ED crowding index was 32, and the coronary artery calcium score was 18.

Table 1 summarizes the clinical characteristics, timing of ED visits, and ED crowding indices. The discrepancy group had a significantly higher median age than the agreement group (67 years [IQR, 58–75] vs. 58 years [IQR, 45–69]; *P* < 0.001). There was no significant difference in the sex distribution between the two groups (agreement: 364 men [53.2%]; discrepancy: 51 men [62.2%]; *P* = 0.123). Patients in the discrepancy group were more likely to have a history of known CAD (15.9% [13/82] vs. 5.4% [37/684]; *P* < 0.001) and hypertension (57.3% [47/82] vs. 38.7% [265/684]; *P* = 0.001) compared to the agreement group. A significantly greater proportion of patients in the discrepancy group were categorized as high-risk by HEART score (54.9% [45/82] vs. 31.9% [218/684]; *P* < 0.001).

Most patients visited the ED at nighttime (84.3%, 646/766), with no significant difference in visit timing between groups. Weekend visits were more frequent in the discrepancy group (57.3% [47/82]), whereas weekday visits were slightly more frequent in the agreement group (55.7% [381/684]; *P* = 0.025). The ED crowding index was significantly lower in the discrepancy group (42.0 [IQR, 37.0–51.2]) than in the agreement group (49.0 [IQR, 39.7–56.2]; *P* = 0.009).

Table 2 presents CCTA findings, CAC scores, image quality, and radiation dose.

Based on final reports, significant CAD was more common in the discrepancy group (41.5% [34/82] vs. 22.8% [156/684]; *P* < 0.001). CAC scores were also higher in the discrepancy group (75.5 [IQR, 22.4–280.0] vs. 0 [IQR, 0–56.5]; *P* < 0.001), with a greater proportion having positive CAC scores (80.5% [66/82] vs. 41.1% [281/684]; *P* < 0.001).

Additionally, fair or poor image quality ratings were more frequent in the discrepancy group (28.0% [23/82] vs. 22.2% [152/684]; *P* = 0.048). No cases in either group were rated as having unassessable image quality. No significant differences were observed in CT scanner type or total dose length product between groups.

**Table 1:** Patient characteristics

	Total (*n* = 766)	Agreement (*n* = 684)	Discrepancy (*n* = 82)	*P* value
Age (year)	59 [47–70]	58 [45–69]	67 [58–75]	< 0.001‡
Men	415 (54.2)	364 (53.2)	51 (62.2)	0.123
Known CAD	50 (6.5)	37 (5.4)	13 (15.9)	< 0.001
Hypertension	312 (40.7)	265 (38.7)	47 (57.3)	0.001
Diabetes	130 (17.0)	110 (16.1)	20 (24.4)	0.058
Hypercholesterolemia	111 (14.5)	95 (13.9)	16 (19.5)	0.172
Ever-smoker	103 (13.4)	87 (12.7)	16 (19.5)	0.088
BMI (kg/m^2^)	23.6 [21.5–25.8]	23.5 [21.5–25.8]	23.7 [22.0–26.1]	0.415‡
Family history of CAD	31 (4.0)	30 (4.4)	1 (1.2)	0.239†
Troponin (pg/mL)	7 [3–20]	6 [3–18]	8 [6–31]	0.001‡
Mean heart rate (beats/min)	65 [58–75]	65 [58–75]	64 [58–75]	0.665‡
HEART score				< 0.001
0–3 (Low risk)	96 (12.5)	95 (13.9)	1 (1.2)	
4–6 (Moderate risk)	407 (53.1)	371 (54.2)	36 (43.9)	
7–10 (High risk)	263 (34.3)	218 (31.9)	45 (54.9)	
Time of ED visit				0.338
Daytime (7:00 AM to 6:00 PM)	120 (15.7)	105 (15.4)	15 (18.3)	
Nighttime (6:00 PM to 7:00 AM)	646 (84.3)	579 (84.6)	67 (81.7)	
Day of ED visit				0.025
Weekdays	416 (54.3)	381 (55.7)	35 (42.7)	
Weekends	350 (45.7)	303 (44.3)	47 (57.3)	
ED crowding index		49.0 [39.7–56.2]	42.0 [37.0–51.2]	0.009‡
<40	211 (27.5)	173 (25.3)	38 (46.3)	< 0.001
≥40	555 (72.5)	511 (74.7)	44 (53.7)	

Note—Data are presented as number (percentage), or median [interquartile range]

Continuous variables were compared using ‡Mann-Whitney U test

Categorical variables were compared using chi-squared test or †Fisher’s exact test

CAD, coronary artery disease; BMI, body mass index; ED, emergency department

**Table 2:** CCTA findings based on final reports and CT-related parameters

	Total (*n* = 766)	Agreement (*n* = 684)	Discrepancy (*n* = 82)	*P* value
CAD-RADS category				< 0.001
0	359 (46.9)	352 (51.5)	7 (8.5)	
1	133 (17.4)	124 (18.1)	9 (11.0)	
2	84 (11.0)	52 (7.6)	32 (39.0)	
3	84 (11.0)	62 (9.1)	22 (26.8)	
4 A	73 (9.5)	62 (9.1)	11 (13.4)	
4B	18 (2.3)	18 (2.6)	0 (0)	
5	15 (1.9)	14 (2.0)	1 (1.2)	
Significant CAD	190 (24.8)	156 (22.8)	34 (41.5)	< 0.001
CAC score	0 [0.0–87.1]	0 [0.0–56.5]	75.5 [22.4–280.0]	< 0.001‡
CAC score category				< 0.001
0	419 (54.7)	403 (58.9)	16 (19.5)	
> 0, ≤ 100	164 (21.4)	133 (19.4)	31 (37.8)	
> 100, ≤ 400	106 (13.8)	84 (12.3)	22 (26.8)	
> 400	77 (10.1)	64 (9.4)	13 (15.9)	
Image quality				0.048
Excellent	21 (2.7)	20 (2.9)	1 (1.2)	
Good	570 (74.4)	512 (74.9)	58 (70.7)	
Fair	159 (20.8)	141 (20.6)	18 (22.0)	
Poor	16 (2.1)	11 (1.6)	5 (6.1)	
Scanner				0.144†
EVO	280 (36.6)	244 (35.7)	36 (43.9)	
Force	486 (63.4)	440 (64.3)	46 (56.1)	
DLP (mGy*cm)	127.2 [81.0–217.1]	124.2 [80.8–206.9]	149.0 [83.1–275.7]	0.246‡

Note—Data are presented as number (percentage), or median [interquartile range]

Continuous variables were compared using ‡Mann-Whitney U test

Categorical variables were compared using chi-squared test for trend or †Fisher’s exact test

CAD-RADS, coronary artery disease reporting and data system; CAD, coronary artery disease; CAC, coronary artery calcium; DLP, dose length product

### Factors associated with discrepancies

The optimal ED crowding index cutoff was 40, based on the receiver operating characteristic curve (AUC = 0.59; sensitivity = 46.3%; specificity = 74.7%).

Table 3 presents the results of logistic regression analyses for factors associated with discrepancies. In univariable analyses, HEART score, ED visit day, ED crowding index, and CAC score were significantly associated with discrepancies.

**Fig. 2:**
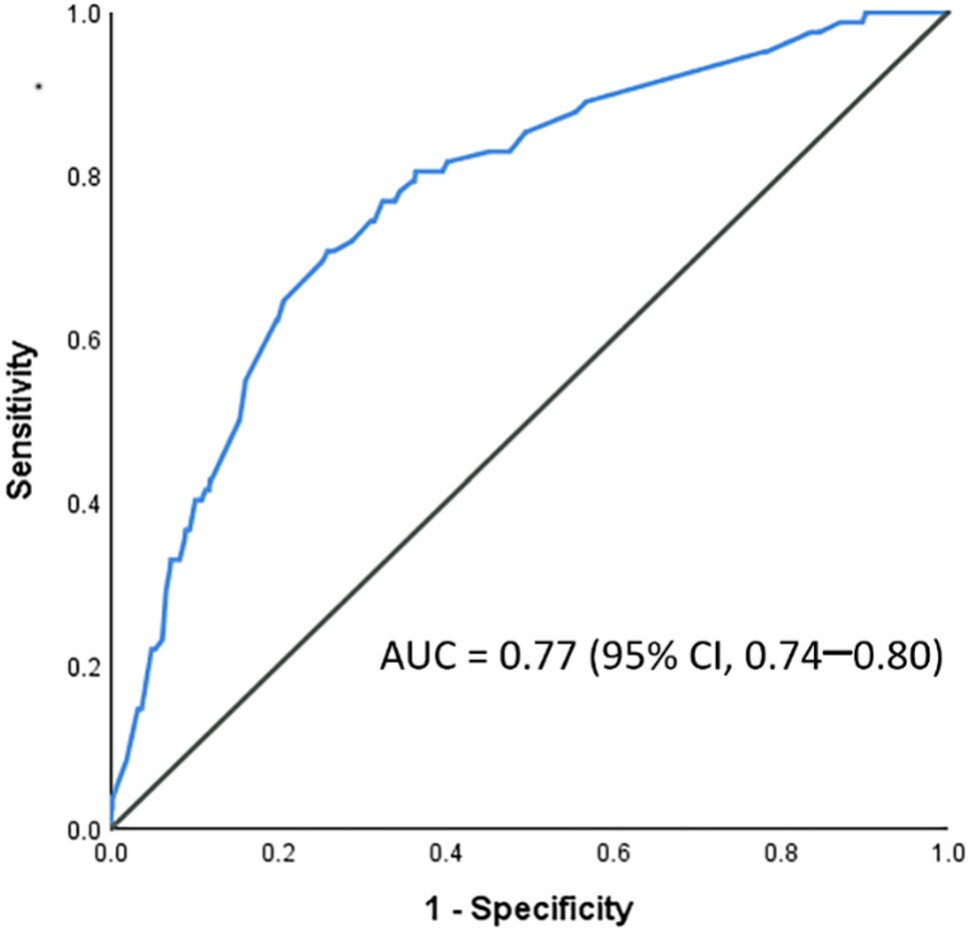
Receiver operating characteristic (ROC) curve of the multivariable logistic regression model for predicting discrepancies between preliminary and final coronary CT angiography interpretations

In multivariable analysis, ED crowding index < 40 (adjusted OR, 2.06; 95% CI: 1.24–3.41; *P* = 0.005) and presence of CAC were independently associated with increased discrepancies. Using CAC score of 0 as the reference, adjusted ORs for discrepancy were 4.56 (95% CI: 2.37–8.76; *P* < 0.001) for scores > 0 and ≤ 100; 4.79 (95% CI: 2.30–9.95; *P* < 0.001) for scores > 100 and ≤ 400; 3.69 (95% CI: 1.61–8.41; *P* = 0.002) for scores > 400. The AUC of the multivariable logistic regression model was 0.77 (95% CI: 0.74–0.80) (Fig. 2).

**Table 3:** Logistic regression analyses for factors associated with discrepancy

	Univariable analysis		Multivariable analysis	
Variable	OR (95% CI)	*P* Value	Adjusted OR (95% CI)	*P* Value
HEART score				
0–3 (Low risk)	Reference		Reference	
4–6 (Moderate risk)	9.37 (1.27–69.21)	0.028	5.75 (0.76–43.37)	0.089
7–10 (High risk)	18.89 (2.56–139.10)	0.004	7.32 (0.95–56.28)	0.056
Time of ED visit				
Daytime (7:00 AM to 6:00 PM)	Reference			
Nighttime (6:00 PM to 7:00 AM)	0.74 (0.41–1.32)	0.323		
Day of ED visit				
Weekdays	Reference		Reference	
Weekends	1.69 (1.06–2.68)	0.026	1.47 (0.89–2.43)	0.132
ED crowding index				
≥40	Reference		Reference	
<40	2.59 (1.62–4.13)	< 0.001	**2.06 (1.24–3.41)**	**0.005**
Mean heart rate				
≤65 beats/min	Reference			
>65 beats/min	0.98 (0.62–1.55)	0.926		
Image quality				
Excellent	Reference			
Good	2.27 (0.30–17.19)	0.429		
Fair	2.55 (0.32–20.18)	0.374		
Poor	9.09 (0.94–87.96)	0.057		
Calcium score				
0	Reference		Reference	
> 0, ≤ 100	6.77 (3.66–12.80)	< 0.001	**4.56 (2.37–8.76)**	**< 0.001**
> 100, ≤ 400	6.73 (3.44–13.35)	< 0.001	**4.79 (2.30–9.95)**	**< 0.001**
> 400	5.28 (2.43–11.55)	< 0.001	**3.69 (1.61–8.41)**	**0.002**

Note—CI, confidence interval; ED, emergency department

### Diagnostic performance and clinical outcomes

**Fig. 3:**
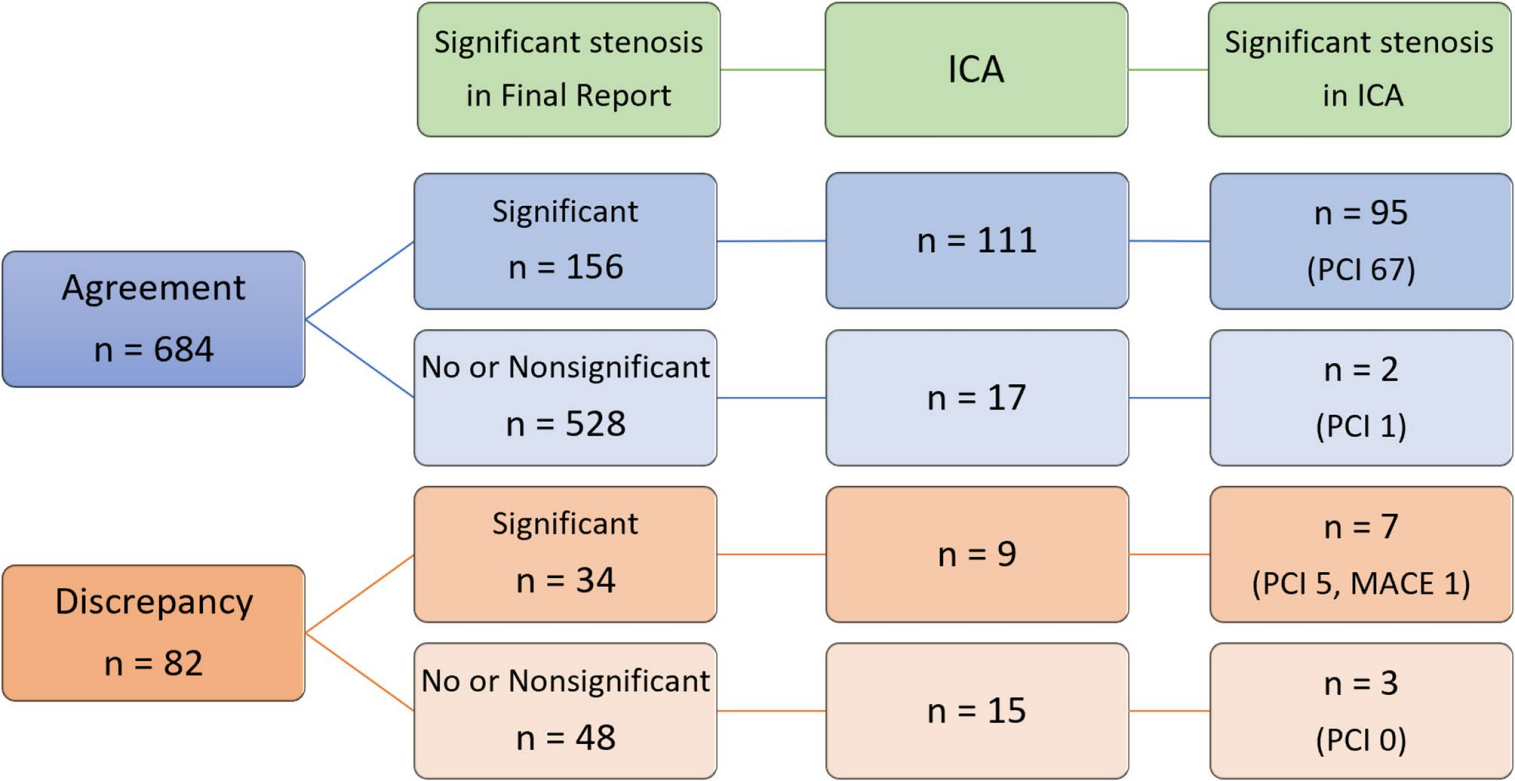
Patient flowchart of ICA procedures and outcomes

A total of 152 patients (19.8%, 152/766) underwent ICA (Fig. 3).

In the agreement group, 156 patients were assessed as having significant stenosis by both residents and cardiac radiologists, and 111 underwent ICA, with an appropriate ICA rate of 85.6% (95/111). The remaining 528 agreement group patients were interpreted as having no or non-significant CAD in both preliminary and final reports, among whom 17 (3.2%) underwent ICA. Of these, only 2 were confirmed to have significant stenosis.

In contrast, in the discrepancy group, 48 patients were initially interpreted as having significant stenosis by residents but reclassified as non-significant in final reports. Of these, 15 underwent ICA, and only 3 were found to have significant stenosis, resulting in an appropriate ICA rate of 20.0% (3/15). Unnecessary ICA rate was significantly higher in the discrepancy group (80.0%, 12/15) compared to the agreement group (14.4%, 16/111; *P* < 0.05).

Additionally, 9 patients (26.5%, 9/34) in the discrepancy group, initially assessed as not having significant CAD, underwent ICA based on clinical judgement and the following day’s final reports indicating significant stenosis. Of these, 7 had significant stenosis and 5 subsequently underwent PCI. One of these patients was admitted to the coronary care unit 19 days after CCTA with unstable angina.

Agreement and Discrepancy groups were divided based on whether the final reports indicated significant stenosis or not. Each group was then subdivided according to whether ICA was performed and whether significant stenosis was confirmed by ICA. PCI and MACE events are noted where applicable.

ICA, invasive coronary angiography; CCTA, coronary computed tomography angiography; IQR, interquartile range; PCI, percutaneous coronary intervention; MACE, major adverse cardiovascular events.

Using ICA results as the reference standard, the final reports had higher sensitivity, specificity, positive predictive value, negative predictive value, and accuracy than preliminary reports (Table 4). Generalized estimating equation analysis confirmed that final reports had significantly better diagnostic accuracy (*P* = 0.003).

**Table 4:** Diagnostic performance of preliminary report and final report

	Preliminary report	Final report
Sensitivity (%)	91.6 (84.8–95.5)	95.3 (89.5–98.0)
Specificity (%)	37.8 (25.1–52.4)	60.0 (45.5–73.0)
PPV (%)	77.8 (69.58–84.2)	85.0 (77.5–90.3)
NPV (%)	65.4 (46.2–80.6)	84.4 (68.2–93.1)
Accuracy (%)	75.7 (68.3–81.8)	84.9 (78.3–89.7)

Note—Data are presented as percentages (95% confidence intervals)

PPV, positive predictive value; NPV, negative predictive value

## Discussion

In this study, the overall discrepancy rate between preliminary and final reports on CCTA performed in the ED was 10.7% (82/766). Both ED crowding index < 40 and positive CAC scores were independently associated with discrepancies, with adjusted ORs of 2.06 and 3.69–4.79, respectively.

This discrepancy rate is notably higher than previously reported major discrepancy rates across various types of diagnostic imaging modalities, ranging from 0.1 to 10% [2, 4–17]. This highlights the challenges of interpreting CCTA compared to imaging of other anatomical regions. Proficiency in CCTA interpretation demands not only adequate experience but also in-depth knowledge of cardiac anatomy and CAD. Recognizing this, current guidelines emphasize the importance of adequate case exposure to achieve diagnostic proficiency [26–28].

Beyond the intrinsic difficulty of CCTA interpretation, external environmental factors in the on-call and ED settings might also contribute to diagnostic errors. Overnight shifts, extended work hours on weekends, and heavy caseloads may impair diagnostic performance by increasing fatigue and reducing the time available for thorough interpretation [29, 30]. Additionally, ED patients often present with comorbid conditions that contraindicate beta-blockers or nitroglycerin, making it difficult to obtain optimal image quality [31]. Conditions such as elevated heart rate or respiratory distress can cause motion artifacts, further degrading image interpretability. In our study, there was no significant difference in mean heart rate between the agreement and discrepancy groups (65 bpm vs. 64 bpm, *P* = 0.665), suggesting that heart rate alone may not fully explain the differences in image quality. This implies that other factors—such as respiratory motion artifacts, high CAC burden, or patients’ body habitus—may have contributed to the perceived image quality and affected diagnostic performance.

Our study highlights specific risk factors associated with discrepancies. Notably, CAC was an independent contributor. Severe CAC can cause blooming artifacts, which obscure accurate lumen assessment and lead to both overestimation and underestimation of stenosis [32, 33]. Interestingly, the odds of a discrepancy were higher in patients with mild to moderate CAC (OR, 4.56–4.79) than in those with severe CAC (OR, 3.69). This finding may suggest that reader experience plays a particularly important role in cases with lower plaque burden, whereas severe calcification is challenging even for experienced readers. This suggests that narrowing the experience-related accuracy gap in cases of lower plaque burden may represent an important focus for CCTA interpretation training. Moreover, artificial intelligence-assisted image reconstruction techniques such as calcium deblooming algorithms, which allow for 24/7 real-time operation, may offer particular benefits in the ED setting [34].

We also found that a lower ED crowding index was associated with a higher discrepancy rate. ED crowding involves multiple factors, and evidence suggests that output factors (e.g., bed availability) have a greater impact on crowding than input factors (e.g., patient volume) or throughput factors (e.g., triage process). This is particularly evident in tertiary hospitals with streamlined and expedited procedural workflows but persistent shortages of inpatient beds [35, 36]. In overcrowded EDs, delayed output leads to longer wait times for new patients and fewer new imaging studies being performed [37]. Such a slowdown in imaging studies may reduce fatigue among on-call residents and allow more time for accurate interpretation, paradoxically associating a higher crowding index with a lower error rate. In our supplementary analysis, we found a weak inverse correlation between the ED crowding index and the number of imaging studies interpreted during a single on-call shift (Spearman’s rho = -0.121; *P* < 0.001). However, since the ED crowding index reflects the number of patients at the arrival time, future studies should explore the relationship between image volume around the time of arrival and diagnostic accuracy, which may better reflect the true impact of crowding on interpretation error.

Time and day were not independently associated with discrepancy rates. Previous studies have suggested that errors are more common during the late-night hours because of fatigue and sleep deprivation [38, 39]; whereas other studies report no significant effect related to timing [40]. In our institution, most off-hour imaging was performed at night, with only a limited number of cases during weekend day shifts. Therefore, it was difficult to fully assess the impact of time-of-day on discrepancies. A subgroup analysis limited to weekend or holiday data, which includes a broader distribution of working hours, may provide additional insights.

This study has several limitations. First, because the management of patients presenting with acute chest pain is based not only on CCTA findings but also on a range of clinical information, the impact of CCTA discrepancy on clinical outcomes—such as inappropriate discharge or delayed treatment—cannot be isolated with certainty. Second, while discrepancies were used as a surrogate for interpretation errors by on-call residents, final reports by attending radiologists may not represent the absolute ground truth, as the possibility of misinterpretation cannot be entirely excluded despite their expertise. Our study aimed to identify systemic factors influencing discrepancies beyond individual reader performance, and our findings support the role of CAC and ED crowding in this context. Lastly, the generalizability of our findings may be limited by our single-center setting and institution-specific reporting workflows. Future multi-center studies may provide broader insight into variability across different ED environments and training programs.

## Conclusions

This study highlights a significant discrepancy rate between preliminary and final CCTA reports in the ED and identifies a lower ED crowding index and positive CAC scores as independent predictors of such discrepancies.

## Data Availability

The datasets used and/or analyzed during the current study are available from the corresponding author on reasonable request.

## Abbreviations

CCTA: Coronary computed tomography angiography
ED: Emergency department
CAD: Coronary artery disease
ICA: Invasive coronary angiography
ECG: Electrocardiogram
CAD-RADS: Coronary artery disease reporting and data system
CAC: Coronary artery calcium
PCI: Percutaneous coronary intervention
MACE: Major adverse cardiovascular events
IQR: Interquartile range
OR: Odds ratio
CI: Confidence interval
AUC: Area under the receiver operating characteristic curve
